# Development of patient-tailored preoperative assessment of percutaneous vertebroplasty

**DOI:** 10.3389/fsurg.2024.1444817

**Published:** 2024-10-24

**Authors:** Yian Lu, Qunhua Jiang

**Affiliations:** ^1^ Department of Orthopedics, Shanghai Fengxian District Central Hospital, Shanghai Jiao Tong University Affiliated Sixth People’s Hospital South Campus, Shanghai, China; ^2^ Department of Nursing, Shanghai Fengxian District Central Hospital, Shanghai Jiao Tong University Affiliated Sixth People’s Hospital South Campus, Shanghai, China

**Keywords:** percutaneous vertebroplasty, bone cement flow, osteoporotic vertebral compression fractures, finite element, fluid flow, porous material

## Abstract

Percutaneous vertebroplasty (PVP), a minimally invasive surgery technique, has become the common treatment for osteoporotic vertebral compression fractures (OVCF). The complications of PVP will lead to severe damage to spinal neuro systems due to bone cement leakage. A patient tailored preoperative assessment approach was developed to reduce the risks of complications in this study. The porcine OVCF model was fabricated to mimic the patient vertebral fracture *in vitro* using decalcification process. The 3D reconstructed model based on the imagological examination data acquired from the porcine vertebral bone was implemented for finite element (FE) simulation. The vertebral body with bone cement injected was scanned using CT for comparison with the finite element simulation results. This study showed a practical method for predicting the flow of bone cement in OVCF, which enabled the surgeons to evaluate the bone cement flow during preoperative assessment to reduce the cement leakage risks.

## 1 Introduction

Percutaneous vertebroplasty (PVP) is a minimally invasive surgery technique, which has been widely used for patients suffering osteoporotic vertebral compression fractures (OVCF) (1–3). The surgical procedure involves percutaneous injection of liquid polymethyl methacrylate (PMMA) bone cement into the fractured vertebra under x-ray image guidance through a large diameter needle, where curing reaction generates heat and transforms the liquid PMMA into solid state that restores the strength and reduce the pain (1, 3–7). The polymerization of the PMMA is a slow heat generation process, which results in the temporally rise of the temperature surround the injected bone cement (8).

One of the common complications is the cement leakage (9, 10). The heat generated from the extravasation of cement will lead to thermal damage to spinal neuro system, which includes paraplegia (11), pulmonary cement embolism (12), neurologic deficits (13) and cardiac perforation (14). Greater injection volume of the cement is one of the factors that increase the risk of the leakage (15, 16), clinical practice tends to become conservative on cement injection volume, which will also lead to insufficient cement distribution that results in intravertebral instability (17) and unrelieved pain (18). Preoperative planning the appropriate cement volume and its distribution will facilitate the surgeons to minimize the cement leakage risk while achieving the satisfactory postoperative recovery of the fractured vertebra.

The flow of the bone cement is influenced by the bone configuration, injection techniques and the cement properties (19). The outer surface of vertebra is formed with solid cortical bone. The inner vertebra body consists of porous cancellous bones (20). The animal experiments have shown that bone loss occurred in cancellous bone and less regional dependent for aged specimens (21). During the PVP intervention, a needle is inserted through the cortical bone into the cancellous bone, followed by PMMA injection through the needle (22). The injection position and cement flow behavior in the cancellous bone determine the cement distribution (23). To predict the bone cement flow in cancellous bones, generic models and experiments have been carried out to investigate the high viscosity cement flow behaviors in porous materials (23, 24). The cylindrical open-porous aluminum foam model was used to represent the cancellous bone for experiment validation (23). The porosity of the aluminum foam was identical for all experiments, which did not reflect the osteoporosis and bone loss in the cancellous bones. The multiphasic finite element (FE) model was developed to predict the PMMA cement flow in the simplified vertebra cancellous bone (24). Without considering the actual geometry factors that influencing the PVP operation, such as the shape of the vertebra and possible needle insertion positions, this model was not able to provide sufficient information for preoperative assessment for the surgeons.

In order to estimate the bone cement leakage, the cement injections were carried out to evaluate the vertebral operation based on the porous material (25, 26). To investigate the bone cement flow characteristics in the cancellous bones, the porous materials used to represent the human cancellous bones in experiments, including the cylindrical or cubical aluminum foams (23, 27) and animal bones (28, 29). The bone cement infiltration of the cancellous bone was the major phenomena that the surgeons were interested in, which was directly related to the bone cement distribution in the cancellous bones. The porcine spine was widely used for vitro biomechanical studies. The vertebral bone osteoporosis models were developed using EDTA to alter the bone mineral density (30).

In order to evaluate the effects of bone loss and injection parameters, the preoperative assessment tool requires the ability to evaluate actual properties of the patients’ vertebral body, such as geometry and fracture locations. Our aim of this study is to develop the process of predicting the bone cement distribution for osteoporosis vertebra fractures. FE model can be developed to estimate the flow behaviors of the cement. The experiments of bone cement injection were carried out to evaluate and validate the FE model, by which the accurate preoperative assessment approach was developed for surgeons to evaluate and tailor the patient-specific operation plans.

## 2 Methods

### 2.1 Sample preparation

The lumbar vertebrae were obtained from commercially available fresh-frozen adult pigs. All specimens had their muscles, ligaments, and periosteum removed. The vertebrae were detached from the spine at the intervertebral disk location, resulting in 25 individual vertebrae. The specimens were sorted into 5 groups in a random manner. All specimens underwent embalming treatment using a 10% formaldehyde solution. Excluding the control group, the samples were immersed in containers filled with EDTA decalcification solution (18.3%, Fulin) with anterior walls facing downward and stored at room temperature following embalming. EDTA was replenished regularly. To facilitate the absorption of EDTA into the cancellous bone in the vertebral body's center, 3 mm diameter holes were drilled at the pedicle on both sides. EDTA was filled daily into the hole as part of the decalcification treatment procedure. The decalcified vertebrae were subjected to mechanical compression using a universal dynamometer from Wance, Shenzhen, China. The compression contact surfaces were the intervertebral disks to prevent radial forces.

### 2.2 Data collection

The computed tomography (CT) scan of the swine vertebral bones was acquired using CT scanner (uCT51 United Imaging, Shanghai, China) with the resolution of 1.2 mm. The CT DICOM-format data were imported to Mimics (Materialise Software, Belgium) for 3D reconstruction. The scan data of vertebra were extracted to generate the 3D model and for bone density evaluation for this study. The decalcification procedure took 12 weeks to complete. CT scans were conducted on swine vertebral bodies at five stages: prior to decalcification, 1 week, 2 weeks, 4 weeks and 12 weeks after decalcification.

### 2.3 Cement injection

The injection of PMMA bone cement was carried out using an inhouse-developed electric motor driven device. The device consisted of a brushless inductive motor, a gearbox, a pushrod, a force transducer, a controller and a syringe adaptor. The piston of the syringe was replaced by the pushrod, which was connected to the force transducer. The forces acting on the pushrod were measured and recorded, which was converted to the pressure as the area of the piston was measured prior to the experiment. The built-in encoder of the electric motor provided the accurate control of the rotating angle, which was converted to the volume control. The accuracy of the volume control was 0.06 ml for the bone cement injection. The syringe adaptor was compatible to the clinical bone cement injector (Dragon Crown Co. Ltd, Shandong, China) along with other PVP operation instruments, such as needles, which did not require the surgeons to alter the operation procedure A graphic user interface (GUI) was developed for experiment control and data recording purposes.

The needles were inserted into the swine vertebral bones via bilateral transpedicular route, which was the most common clinical approach (7). The bone cement was prepared prior to the experiment and injected into the vertebral bones using the inhouse-developed device. The effective volumes of 2 ml, 3 ml and 4 ml were injected into three vertebral bones under the velocity of 0.2 ml/s for each side, which were measured and recorded with subtracting the needle residual volume of 1.8 ml, which was measured prior to the experiment. The injecting pressures were simultaneously measured and recorded during the injection procedure using the force transducer integrated in the device.

After the injected bone cement was settled and polymerized, the geometry and distribution of the vertebral bones with bone cement was acquired by CT scanning. The CT images were processed in Geomagic software to extract the morphology of the dispersion of the injected bone cement.

### 2.4 FE simulation

To validate the result and to investigate the bone cement flow characters, the FE numerical modelling was carried out based on the experiment results. The simulation results were compared with the experiment data considering the free flow pathway. The improved model with same configuration except the existence of the free flow pathway was developed afterwards, so that the feasibility to PVP preoperational evaluation was assessed.

The bone cement flow in cancellous bone was modelled as two-phase incompressible fluid flow in the porous material, which was described by the Darcy's law in mathematic expression (25)

(1) $$\rho \frac{\partial \varepsilon_{p}}{\partial t}+\nabla \cdot (\rho u)=0,$$

where *ρ* was the cement density, *ɛ_p_* the porosity and **u** the velocity, which was defined as

(2) $$u=-\frac{\kappa }{\mu }\nabla p,$$

where *κ* was the permeability, *μ* the viscosity and *p* the pressure.

Considering two incompressible fluid phases flow, i.e., bone cement and marrow, in the simulations, the density was defined to be algebraic sum proportional to the phase volumetric portion, which was eressed as

(3) $$\rho =s_{M}\rho_{M}+s_{C}\rho_{C},$$

where *s* was the volumetric percentages of the fluids. The subscript *M* and *C* denoted the marrow and cement, respectively. The viscosity was calculated by

(4) $$\frac{1}{\mu }=\frac{s_{M}}{\mu_{M}}+\frac{s_{C}}{\mu_{C}}.$$

The flow behavior of PMMA can be described by Equations 1–4. The densities for the PMMA and marrow were 1,200 kg/m^3^ and 1,060 kg/m^3^ (24), respectively. The typical viscosity value of 400 Pa·s was used for PMMA bone cement (27) and 0.4 Pa·s for marrow (21). The permeability and porosity of the cancellous bone were set to 8.47 × 10^−10^ m^2^ (23) and 91% (31), respectively.

The injection velocity of the PMMA was set to 0.2 ml/s according to the experimental data.

## 3 Results

### 3.1 Experiment results

**Figure 1:**
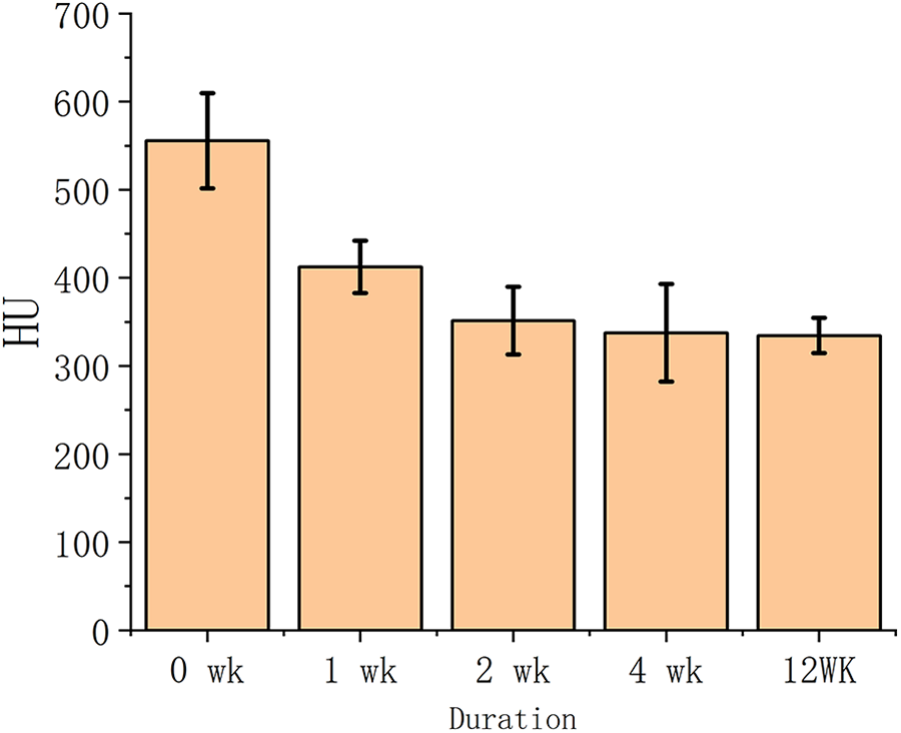
The cancellous bone CT image greyscale value vs. EDTA decalcification duration.

The osteoporotic vertebrae decalcified by EDTA were evaluated after CT scan based on the grayscale value of the images. Figure 1 displayed the cancellous bone CT image grayscale measurement of the vertebrae at various decalcification stages. The CT image HU values were measured to represent the cancellous bone density variation regarding to the non-decalcification treated specimens. The results showed that the decalcification procedure with EDTA effectively reduced the bone density of cancellous bone. Initially, EDTA showed greater efficacy than it did at a later stage of the process. After a 12-week treatment period, there was minimal variation in the measurements between the specimens treated for 0, 1 2, 4 and 12 weeks.

**Figure 2:**
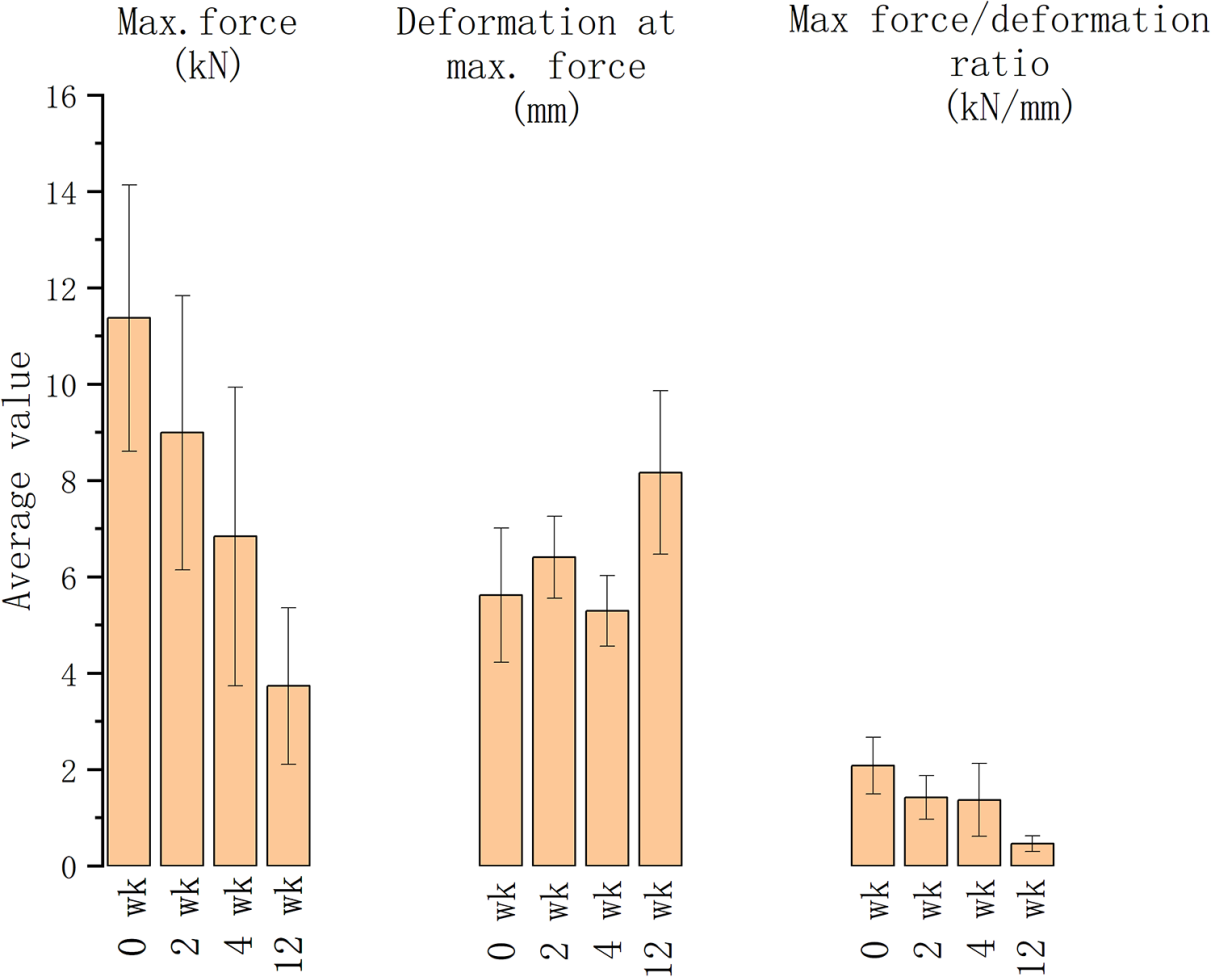
The typical CT image of vertebral bone after decalcification treatment.

The prolonged decalcification treatment decreased the strength and hardness of the vertebrae and lowered bone density. The strength and hardness of cortical bone are significantly higher than those of cancellous bone, resulting in a larger decalcification impact from EDTA on cortical bone compared to cancellous bone. Figure 2 depicted the typical vertebral bones after different decalcification duration.

**Figure 3:**
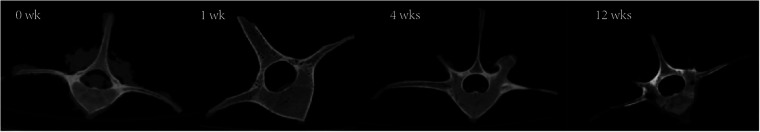
The maximum force, deformation at maximum force and force/deformation ratio of the vertebrae with different decalcification durations.

The vertebrae treated for 0, 1, 4 and 12 weeks were compressed using a universal dynamometer to establish the vertebral compression fractures. The maximum forces decreased as the decalcification duration rose, shown in the maximum force and deformation reduction (as depicted in Figure 3). The degree of compression fracture deformation rose in association with longer decalcification treatment durations. As the EDTA decalcification duration of treatment increased, its effect on decalcifying cortical bone turned more pronounced, leading to a decrease in the vertebral body's resistance to external pressure. The impact of EDTA on decalcification of cancellous bone reduced while the duration of exposure increased.

### 3.2 Simulation results

**Figure 4:**
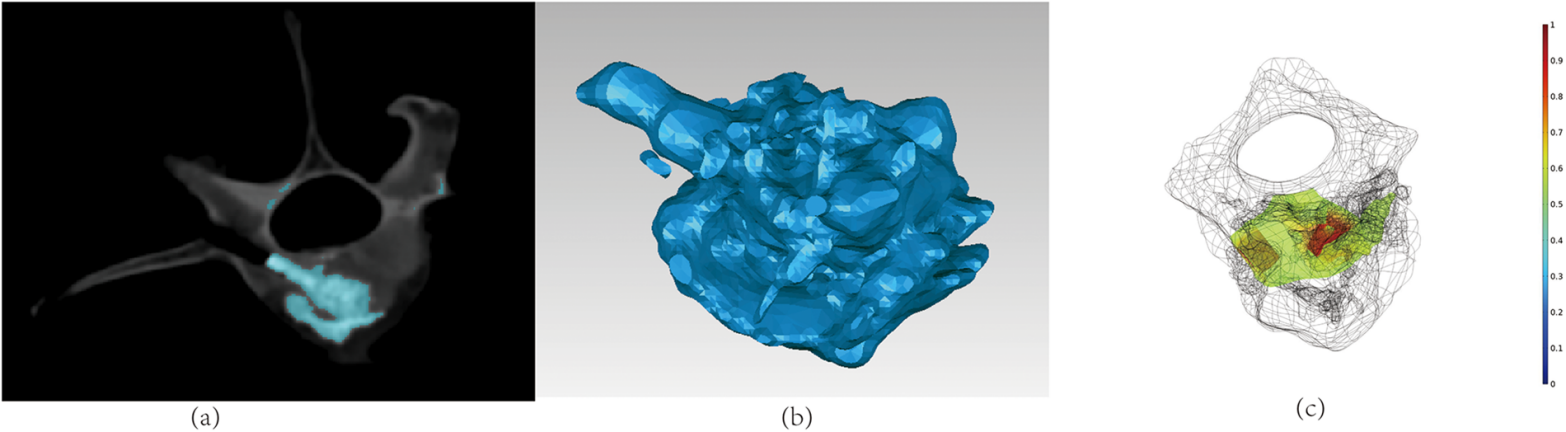
The comparison of experiment and simulation of bone cement flow in fractured cancellous bone: **(a)** the CT image of the vertebral body after the bone cement injection; **(b)** the 3D reconstructed bone cement dispersion of the experiments; **(c)** the simulated result of bone cement dispersion.

A three-dimensional reconstruction of the vertebral body model following a compressive fracture was developed using a CT scan. The model was reconstructed prior to and after the injection of bone cement, incorporating cortical bones, cancellous bones, and cancellous bone fractures. The PMMA bone cement and marrow flow in the fractured vertebral cancellous bone was simulated based on the reconstructed 3D model prior to the bone cement injection. The cortical bones confined the flow boundary of bone cement in the FE simulation model. The fractures in the cancellous bones were replicated using a cancellous bone model with extremely low density and large porosity, so that nearly free flow was allowed in the fractures. The simulated PMMA bone cement dispersion was compared with the CT reconstruction results in the cancellous bone. Based on Figure 4, the simulated results were substantially consistent with the experimental data. The bone cement flow model in the cancellous bone correlates with the experimental flow direction, demonstrating the method's ability to predict the bone cement behavior in compressive fracture vertebral bodies.

## 4 Discussion

PVP is currently the most popular treatment for osteoporotic vertebral compression fractures. Rapid rehabilitation is crucial in obese patients with a concurrent high body mass index (BMI) to reduce problems following spine injury (32). The major complication of PVP was the bone cement leakage, which resulted in the nerve root injury (9, 15, 33). Most surgeon tends to reduce the injected bone cement volume to avoid the risk of bone cement leakage. This may lead to insufficient pain relief (17). The correct injection volume needs to be determined based on specific patient data, as the fracture location varies depending on the patients. The approach developed in this study enabled the surgeons to evaluate and preview the critical volume for bone cement leakage based on the preoperative CT data and FE simulations.

Decalcified porcine vertebrae were used for generating osteoporotic vertebral models. EDTA was determined to be successful in carrying out decalcification by assessing vertebral bone density after decalcification based on CT image grayscale measurement. After 4 weeks of decalcification, there was a considerable reduction in bone mineral density (BMD) in cancellous bone. EDTA was successful in providing continuous decalcification in cortical bone, aligning with findings from other research (30). The mechanical properties of the vertebral body are significantly influenced by cortical bone, which is relied upon to support external loads in normal situations. Under typical circumstances, the vertebral body depends on the cortical bone to support external loads. After decalcification of the cortical bone, its ability to carry external loads decreases, which leads to a significant compressive deformation of external loads on cancellous bone. CT scans can reveal ruptures in the cancellous bone of osteoporotic vertebrae when they are under stress, which aligns with the clinical observation. When osteoporotic vertebrae were mechanically compressed after EDTA decalcification, the maximum load force was dramatically decreased, while the deformation was raised. The ratio of maximum load to deformation dramatically increased, demonstrating a notable decrease in the bearing capacity of the vertebral bodies in the osteoporotic model for external loads. The cancellous bone model in this study can be deemed successful.

The finite element simulation model was based on vertebral body construction prior to injection of bone cement and modeling of osteoporotic compression fractures. The bone cement in the simulation model propagates within the cancellous bone. The bone cement tends to flow more towards the fracture location due to its presence, leading to an unevenly distributed dispersion of the bone cement. The simulation results demonstrate that the distribution of bone cement in cancellous bone aligns well with the experimental results, suggesting that its flow behavior conforms to the two-phase flow rule and may be represented by Darcy's law of two-phase flow. The cancellous bone model in this work was created by decalcifying vitro vertebrae, which did not accurately replicate the porous changes in cancellous bone that occur after decalcification *in vivo*, resulting in subtle alterations in porosity. Compression fractures were simulated using materials with a very low porosity. Bone cement exhibits greater mobility and reduced resistance as it flows more readily towards the fractured area. This approach preserves the model's integrity and allows for simulating the flow properties of the bone cement. The simulation result indicated that the free flow path reduced the pressure gradient that drove the injected fluid dispersion in sagittal direction, which validated the FE model according to the CT data.

However, there are several limitations in this study. Due to decalcification in isolated vertebral bodies via EDTA, osteoporosis models cannot effectively simulate changes in porosity. The cancellous bone on the CT scan exhibited a reduction in the overall gray value, rather than an increase in the number of holes. Reconstruction of 3D models based on CT data requires manual intervention, so for clinical applications, automated data processing processes need to be further developed to optimize the process of simulation prediction. Prior studies have demonstrated that the preoperative Femoral Obliquity Angle (FOA) and T1 Pelvic Angle (TPA) are significant prognostic factors in predicting patients’ disability and quality of life following spinal surgery. Additionally, they serve as early indications of potential sagittal plane deformity of the spine (34). Considering characteristics such as FOA and TPA is crucial in the planning of PVP surgery as well.

## 5 Conclusion

The approach developed in this study facilitate a patient-tailored PVP preoperational assessment and evaluation based on the CT data. Compression fractures resulting from osteoporosis have been generated via the decalcification of the porcine vertebra *in vitro*, followed by mechanical compression. The distribution of bone cement within the cancellous bone was simulated using a two-phase flow model, accurately forecasting its flow within the cancellous bone model. The approach developed in this study was able to mimic the bone cement flow in vertebral bone to reduce the risk of bone cement leakage.

## Data Availability

The raw data supporting the conclusions of this article will be made available by the authors, without undue reservation.
